# Supplementary material for: Study of low-dose PET image recovery using supervised learning with CycleGAN
Source: PLoS One. 2020 Sep 4;15(9):e0238455. doi: 10.1371/journal.pone.0238455 (PMC7473560; doi:10.1371/journal.pone.0238455)
Supplement: Attachment — Submitted filename: Response to reviewers.pdf [file pone.0238455.s001.pdf]

*We would like to thank editors and reviewers for their valuable time and very careful assessment of our manuscript. Following are our point-by-point answers to reviewers' comments. Changes in the manuscript are highlighted by the red color.*

PONE-D-19-34960
Study of low-dose PET image recovery using supervised learning with CycleGAN
PLOS ONE

Reviewer #1: REVIEW: "Study of low-dose PET image recovery using supervised learning with CycleGAN"

Comments:
The authors do not substantiate the choice of the CycleGAN architecture (designed for unpaired datasets) on a paired dataset. The benefit of adding a second generator/discriminator pair to transfer an image from the FDPET domain to the LDPET domain is unclear.
*As we know, the CycleGAN model is originally designed for unpaired data and style or feature transfer. However, in recent published studies (Harms et al. 2019), training images are paired by registration or as similar in structure as possible to preserve quantitative pixel values, lead network to focus on mapping details by removing large geometric mismatch, and accelerate training. PET image reconstruction involves complex physical corrections including attenuation, random and scatter corrections, which complicate the noise pattern in the reconstructed PET image. Therefore, there are not only differences in the noise pattern, but also potential differences in the image structure between LDPET and FDPET (The major difference is the noise pattern though). In LDPET denoising, we treated the different noise patterns as features, and the denoising process as the feature transfer. When we trained CycleGAN model in the unsupervised learning fashion, some artifacts of missing structures are observed in about 7% of images.*
*So, the additional supervised learning loss effectively guides the transformation of the model mainly focusing on noise patterns. (More details can be found in Section "Ablation Study", Line 251-263)*

This claim is unproven: "the proposed method is better at preserving image details than the other two methods". The samples shown in figures 7 and 8 seem to support this claim, but a quantitative justification should be given with the proper metrics. Indeed, the NRMSE, SSIM, and PSNR are not adequate perceptual metrics (https://arxiv.org/abs/1801.03924). A perceptual loss (LPIPS) or a mean opinion score measure (MOS) could be used to support this statement.
*Thanks for your suggestion, we added a LPIPS comparison plot for S-CycleGAN, RED-CNN and 3D-cGAN. Our proposed model, S-CycleGAN, could obtain the best perception score. (Figure 9)*

Miscellaneous remarks:

- Typo in the abstract: “S-CylceGAN”
- Typo line 78: “supervise”

*We appreciate the reviewer for helping us to improve grammar and we have revised the manuscript appropriately.*

Reviewer #2: This paper investigates a supervised, cycle-consistent, conditional GAN to map low-dose PET brain images to full-dose images. Reported performance is promising compared to other deep learning frameworks. However, the study has two main limitations that need to be addressed before possible publication.

* A training set (99 samples) and a test set (10 samples) were defined. However, no validation set is mentioned, which is a severe limitation. In particular, it is not clear how the numerous hyperparameters (the alpha, beta and gamma loss weights, the learning rate, Adam optimizer's beta1 and beta2 parameters, the patch size, network hyperparameters, etc.) were set: if the number of hyperparameters is close to the number of test samples (10), then the selection criterion for these hyperparameters is very critical. Also, it is not clear how the stopping criterion was defined. Training is generally stopped when the validation loss is minimal. When is it stopped if no validation set was defined? When the test loss is minimal? Given the small number of samples (109), I recommend to follow a cross-validation strategy on the 99 development samples: the value of hyperparameters (including the number of training epochs) could be defined as those maximizing the cross-validation score. A final model would then be trained on the entire development set using the optimal hyperparameter values.

*Thanks for your suggestion. Since we didn’t have plenty of clinical PET images, we cropped image patches from the original PET images to augment the datasets for both training and testing. Actually, there were 136704 and 15360 patches (i.e. 89 and 9 patients) for training and validation though we didn’t include those details in the previous manuscript (See Section “Datasets”).*

*The hyperparameters basically were derived from the original CycleGAN paper. As for the parameter, $\gamma$, it was determined by experiments in order to get a trade-off between the noise and the bias of SUV in the lesion regions.*

* Each of the four training losses (Adversarial Loss, Cycle Consistency Loss, Identity Loss, Supervised Learning Loss) seem relevant. However, it is not clear how each of them contribute to the overall performance. Note that the alpha, beta and gamma weights are not reliable indicators in themselves. The added value of this paper would be much higher if the impact of each loss function was analyzed independently: this could be done by retraining the system without the loss function to analyze.

*We made an ablation study to demonstrate our design, and results have been included in the manuscript. (Line 251 – 263)*

Minor comments are listed below:

* Other papers address the same task using CycleGANs (for instance "Yang Lei et al. Low dose PET imaging with CT-aided cycle-consistent adversarial networks, Proc. SPIE 11312, Medical Imaging 2020: Physics of Medical Imaging, 1131247 (16 March 2020); https://doi.org/10.1117/12.2549386"). Differences with these papers should be highlighted.
* The expression of the Wasserstein distance should be provided.
* Training details for RED-CNN and 3D-cGAN solution should also be provided. Moreover, methodological differences between these solutions and the proposed solution should be better highlighted.
* Why providing hardware details (NVIDA TITAN GTX GPU) if no computation times are given? Training times and inference times should be provided.

*The references, the expression of Wasserstein distance, training details for RED-CNN, 3D-cGAN, and training and inference time information have been appropriately added.*

Typos:

* applicaions -> applications
* an Machine Learning -> a Machine Learning

*We appreciate the reviewer for helping us to improve grammar and we have revised the manuscript appropriately.*

Reference:

Harms, J., Lei, Y., Wang, T., Zhang, R., Zhou, J., Tang, X., Curran, W.J., Liu, T., Yang, X., 2019. Paired cycle-gan-based image correction for quantitative cone-beam computed tomography. Medical Physics 46, 3998–4009. URL: https://onlinelibrary.wiley.com/doi/abs/10.1002/mp.13656, doi:10.1002/mp.13656.
